# Clinical application of biological markers for treatments of resectable non-small-cell lung cancers

**DOI:** 10.1038/sj.bjc.6602481

**Published:** 2005-03-22

**Authors:** C Huang, D Liu, D Masuya, T Nakashima, K Kameyama, S Ishikawa, M Ueno, R Haba, H Yokomise

**Affiliations:** 1 Second Department Surgery, Faculty of Medicine, Kagawa University, 1750-1, Miki-cho, Kita-gun, Kagawa 761-0793, Japan; 2 Pathology and Host Defense, Faculty of Medicine, Kagawa University, Kagawa, Japan; 3 Department of Pathology, Faculty of Medicine, Kagawa University, Kagawa, Japan

**Keywords:** lung cancer, biological marker, prognosis, immunohistochemistry

## Abstract

We performed a clinical study to identify biological markers useful for the treatment of resectable non-small-cell lung cancers (NSCLCs). In all, 173 patients were studied. By immunohistochemistry, we evaluated the Ki-67 proliferation index, tumour vascularity, thymidylate synthase (TS), vascular endothelial growth factor (VEGF)-A, VEGF-C, and E (epithelial)-cadherin. Concerning the survival of NSCLC patients, tumour vascularity (*P*<0.01), VEGF-A status (*P*=0.03), VEGF-C status (*P*=0.03), and E-cadherin status (*P*=0.03) were significant prognostic factors in patients with stage I NSCLCs. The Ki-67 proliferation index (*P*=0.02) and TS status (*P*<0.01) were significant prognostic factors in patients with stage II–III NSCLCs. In patients with stage II–III NSCLCs, furthermore, the survival of UFT (a combination of tegafur and uracil)-treated patients with TS-negative tumours was significantly better than those of any other patients. Biological markers associated with tumour angiogenesis or metastasis are useful for the detection of aggressive tumours among early-stage NSCLCs. Postoperative chemotherapy might be necessary in such tumours even in stage I. In contrast, tumour proliferation rate and TS status are useful markers for identifying less aggressive tumours in locally advanced NSCLCs. Thymidylate synthase expression is also a useful marker to evaluate responsiveness of UFT-based chemotherapy for these tumours.

Non-small-cell lung cancer (NSCLC) is one of the most common human malignancies with a poor prognosis. Surgical resections play major roles in managing patients with stage I and stage II NSCLCs, and may be used for patients with stage III NSCLCs (Cersosimo, 2002). In addition, various combined-modality therapy, including chemotherapy and radiation therapy, have been assessed for improving the outcome of patients with NSCLCs (Arriagada *et al*, 2004; Machtay *et al*, 2004; Strauss *et al*, 2004; Winton *et al*, 2004). However, the 3-year survival of stage II NSCLC patients was 35–55%, and the 3-year survival of resectable stage III NSCLC patients was 28% (Martin *et al*, 2002). Therefore, a new therapeutic strategy is required for patients with resectable NSCLCs.

Owing to developments in molecular biology, many clinical studies on molecular markers associated with tumour biological behaviour have been performed in human cancers, and these molecular markers, including oncogenes, tumour suppressor genes (Huang *et al*, 1998), metastatic suppressor genes (Adachi *et al*, 1998; Sulzer *et al*, 1998), and angiogenetic factors (Imoto *et al*, 1998; Masuya *et al*, 2001), could be prognostic factors for NSCLC patients. Therefore, to improve the treatment of NSCLC patients, it is important to decide the optimal therapeutic strategy for them according to the tumour biology. Our previous studies demonstrated four biological markers, including thymidylate synthase (TS) (Huang *et al*, 2000), vascular endothelial growth factor-A (VEGF-A), VEGF-C (Nakashima *et al*, 2004), and E (epithelial)-cadherin (Liu *et al*, 2001), to be significant prognostic factors for NSCLC patients. Thymidylate synthase is reported to be related to tumour cell proliferation and responsiveness to 5-FU-based chemotherapy (Navalgund *et al*, 1980; Beck *et al*, 1994). VEGF-A and VEGF-C are members of the VEGF family associated with angiogenesis or lymphangiogenesis (Dvorak *et al*, 1995; Skobe *et al*, 2001). E-cadherin is one of the metastatic suppressor genes (Mbalaviele *et al*, 1996; Sulzer *et al*, 1998).

Therefore, we performed a retrospective clinical study to identify molecular markers useful for the treatment of patients with resectable NSCLCs. We evaluated the intratumoral expressions of these four biological markers: TS, VEGF-A, VEGF-C, and E-cadherin. In addition, we studied the tumour proliferation rate using the Ki-67 index (Gerdes *et al*, 1984; Scagliotti *et al*, 1993) and tumour angiogenesis using CD34 staining (Matsuyama *et al*, 1998).

## MATERIALS AND METHODS

### Clinical characteristics of patients

From January 1995 to December 1997, consecutive NSCLC patients who underwent surgery at the Second Department of Surgery, Faculty of Medicine, Kagawa University were studied. This study was approved by the institutional review board of Kagawa University (14-7, a clinical study of biological markers in NSCLCs). TNM staging designations were made according to the postsurgical pathological international staging system (Mountain, 1997). In total, 173 patients with lung cancer up to stage III were investigated (Table 1). These included 97 patients with stage I NSCLCs, 17 patients with stage II NSCLCs, and 59 patients with stage III NSCLCs. Patients' clinical records and histopathological diagnoses were fully documented. This report includes follow-up data as of 31 March 2004. The median follow-up period for all patients was 77.0±28.9 months.

Regarding methods of surgical resection, a pneumonectomy was performed in 16 patients with stage II–III NSCLCs. A lobectomy was performed in 133 patients: 73 patients with stage I NSCLCs, 15 patients with stage II NSCLCs, and 45 patients with stage III NSCLCs. A segmentectomy was performed in three patients with stage I NSCLCs, and a wedge resection was performed in 21 patients with stage I NSCLCs. Systemic chemotherapy using mitomycin/vinblastin/cisplatin (MVP) was performed in all patients with stage II–III NSCLCs; neoadjuvant chemotherapy in 49 patients, and postoperative adjuvant chemotherapy in 27 patients with nodal metastases. Radiation therapy was performed in 18 patients: 11 patients with T3 or T4 status and seven patients with mediastinal lymph node metastases. 5-FU was administered as UFT (a combination of tegafur and uracil, Taiho Pharmaceutical Co., Tokyo, Japan; 300–400 mg day^−1^ body^−1^) to 107 patients including 53 patients with stage I NSCLCs and 54 patients with stage II–III NSCLCs. Oral administration of UFT was started within 1 month after surgery. UFT was administered for 2 years if there was no recurrence of carcinomas. In patients with recurrences, UFT was given until its oral administration became impossible.

### Immunohistochemistry

The following antibodies were used, along with isotype antibodies as negative controls: a mouse monoclonal antibody for the Ki-67 antigen (MIB-1, DAKO, Glostrup, Denmark) diluted at 1 : 40, a mouse monoclonal antibody for CD34 (NU-4A1, Nichirei Corporation, Tokyo, Japan) diluted at 1 : 10, a rabbit polyclonal antibody for TS (kindly provided by Dr M Fukushima) (Huang *et al*, 2000) diluted at 1 : 500, a rabbit polyclonal antibody for VEGF-A (A-20, Santa Cruz Biotechnology, Santa Cruz, CA, USA) diluted at 1 : 200, a goat polyclonal antibody for VEGF-C (N-19, Santa Cruz Biotechnology) diluted at 1 : 100, and a mouse monoclonal antibody for E-cadherin (HECD-1, Takara, Otsu, Japan) diluted at 1 : 400.

Formalin-fixed paraffin-embedded tissue was cut into 4-*μ*m sections and mounted onto poly-L-lysine-coated slides. Sections were deparaffinised and rehydrated. The slides were then heated in a microwave for 10 min in a 10-*μ*mol l^−1^ citrate buffer solution at pH 6.0, and cooled to room temperature for 20 min. After quenching the endogenous peroxidase activity with 0.3% H_2_O_2_ (in absolute methanol) for 30 min, the sections were treated for 2 h at room temperature with 5% bovine serum albumin to block nonspecific staining. Duplicate sections were incubated overnight with the primary specific antibodies detecting Ki-67, CD34, TS, VEGF-A, VEGF-C, and E-cadherin. Slides were then incubated for 1 h with biotinylated anti-mouse IgG (Vector Laboratories Inc., Burlingame, CA, USA) for Ki-67, CD34, and E-cadherin, biotinylated anti-rabbit IgG (Vector Laboratories Inc.) for TS and VEGF-A, and biotinylated anti-goat IgG (Vector Laboratories Inc.) for VEGF-C. The sections were incubated with the avidin–biotin–peroxidase complex (Vector Laboratories Inc.) for 1 h, and antibody binding was visualised with 3,3′-diaminobenzidine tetrahydrochloride. Lastly, the sections were lightly counterstained with Mayer's haematoxylin. The human colon cancer cell line DLD-1/FrUrd was used as a positive control for the staining of TS. Sections of resected lung tumours known to express VEGF-A, VEGF-C, or E-cadherin were used as positive controls for immunohistochemic staining, respectively.

### Assessment of immunohistochemical staining

All of the immunostained sections were reviewed by two pathologists (M Ueno and R Haba) who had no knowledge of the patients' clinical status. The percentage of carcinoma cells with positive staining for Ki-67 in a given specimen was scored as the Ki-67 proliferation index, and tumours with ⩾25% of Ki-67 proliferation index were classified as high Ki-67 (Scagliotti *et al*, 1993). For microvessel quantification, the three most highly vascularised areas detected by CD34 immunostaining were initially selected under the × 40 field, and a × 200 field (0.785 mm^2^ per field) was used to count vessels in each of these areas. The average of three × 200 field counts was recorded as the intratumoral microvessel density (IMD). Tumours with ⩾90 of the IMD were classified as hypervascular (Masuya *et al*, 2001).

For TS, VEGF-A VEGF-C, and E-cadherin, samples were classified into two groups, positive or negative, with a cutoff value based on the findings of previous reports, respectively. At least 200 tumour cells were scored per × 40 field. All sections were scored in a semiquantitative manner according to the method described previously, which reflects both the intensity and percentage of cells staining at each intensity (McCarty *et al*, 1986). Intensity was classified as 0 (no staining), +1 (weak staining), +2 (distinct staining), or +3 (very strong staining). A value designated the ‘HSCORE’ was obtained for each slide by using the following algorithm: HSCORE=Σ(*I* × PC), where *I* and PC represent staining intensity and the percentage of cells that stain at each intensity, respectively, and the corresponding HSCOREs were calculated separately. Concerning TS expression, when the HSCORE of TS in a given specimen was ⩾30, the sample was classified as TS-positive (Huang *et al*, 2000). Expressions of VEGF-A, VEGF-C, and E-cadherin were classified as follows: when ⩾30% of the carcinoma cells in a given specimen were positively stained for VEGF-A, the sample was classified as VEGF-A-positive (Masuya *et al*, 2001); when ⩾30% of the carcinoma cells in a given specimen were positively stained for VEGF-C, the sample was classified as VEGF-C-positive (Arinaga *et al*, 2003); and when ⩾50% of the carcinoma cells in a given specimen were positively stained for E-cadherin, the sample was classified as E-cadherin-positive (Liu *et al*, 2001).

### Statistical analysis

The statistical differences in the expression of each biological marker in relation to various clinical and pathological parameters were assessed by the *χ*^2^ test. Overall survival was defined as the time from treatment initiation (surgical resection, chemotherapy, or radiation) to the date of death from any cause. The Kaplan–Meier method was used to estimate the probability of overall survival as a function of time, and differences in the survival of subgroups of patients were compared by using Mantel's log-rank test. Multivariate analyses were performed using the Cox regression model to study the effects on survival (Cox, 1972). The validity of the proportional hazards assumption was investigated by graphical methods (Grambsch and Therneau, 1994). All *P*-values were based on two-tailed statistical analysis, and a *P*-value <0.05 was considered to indicate statistical significance.

## RESULTS

### Distribution of biological markers in NSCLCs

Concerning the Ki-67 proliferation index, 117 carcinomas (67.6%) had high Ki-67 (Table 2). The frequency of high Ki-67 tumours was significantly higher in squamous cell carcinomas than in adenocarcinomas (87.9 *vs* 56.4%, *P*<0.01). However, there was no difference in the Ki-67 index in relation to tumour status, nodal status, pathological stage, or neoadjuvant chemotherapy.

With respect to tumour angiogenesis, 93 carcinomas (53.8%) were hypervascular tumours. The frequency of hypervascular tumours was significantly higher in adenocarcinomas than in squamous cell carcinomas (65.3 *vs* 32.8%, *P*<0.01). There was no difference in the IMD according to tumour status, nodal status, pathological stage, or neoadjuvant chemotherapy.

Concerning the intratumoral TS expression status, 93 carcinomas (53.8%) were TS-positive. The frequency of TS-positive tumours was significantly higher in squamous cell carcinomas than in adenocarcinomas (72.4 *vs* 43.6%, *P*<0.01). There was also no difference in TS status according to tumour status, nodal status, pathological stage, or neoadjuvant chemotherapy.

In all, 91 carcinomas (52.6%) were VEGF-A-positive tumours. There was no difference in VEGF-A status in relation to tumour histology or neoadjuvant chemotherapy. However, the frequency of nodal metastases was significantly higher in VEGF-A-positive tumours than in VEGF-A-negative tumours (37.4 *vs* 23.2%, *P*=0.04).

A total of 73 carcinomas (42.2%) were VEGF-C-positive tumours. There was no difference in VEGF-C status in relation to tumour histology, tumour status, nodal status, pathological stage, or neoadjuvant chemotherapy.

In total, 98 carcinomas (56.6%) were E-cadherin-negative. There was no difference in E-cadherin status in relation to tumour histology or neoadjuvant chemotherapy. However, the frequency of nodal metastases was significantly higher in E-cadherin-negative tumours than in E-cadherin-positive tumours (37.8 *vs* 21.3%, *P*=0.02).

### Ki-67 proliferation index in relation to biological markers in NSCLCs

Concerning TS status, the Ki-67 proliferation index was significantly higher in TS-positive tumours than in TS-negative tumours (48.6±29.9 *vs* 35.6±29.8, *P*<0.01). Regarding VEGF-A status, the Ki-67 proliferation index was significantly higher in VEGF-A-positive tumours than in VEGF-A-negative tumours (46.9±31.5 *vs* 37.7±28.6, *P*=0.04). However, there was no difference in the Ki-67 proliferation index in relation to VEGF-C or E-cadherin status.

### IMD in relation to biological markers in NSCLCs

Concerning VEGF-A status, the IMD was significantly higher in VEGF-A-positive tumours than in VEGF-A-negative tumours (118.9±51.6 *vs* 91.1±55.8, *P*<0.01). However, there was no difference in the IMD in relation to TS, VEGF-C, or E-cadherin status.

### Survival of patients with stage I NSCLCs in relation to biological markers

**Figure 1:**
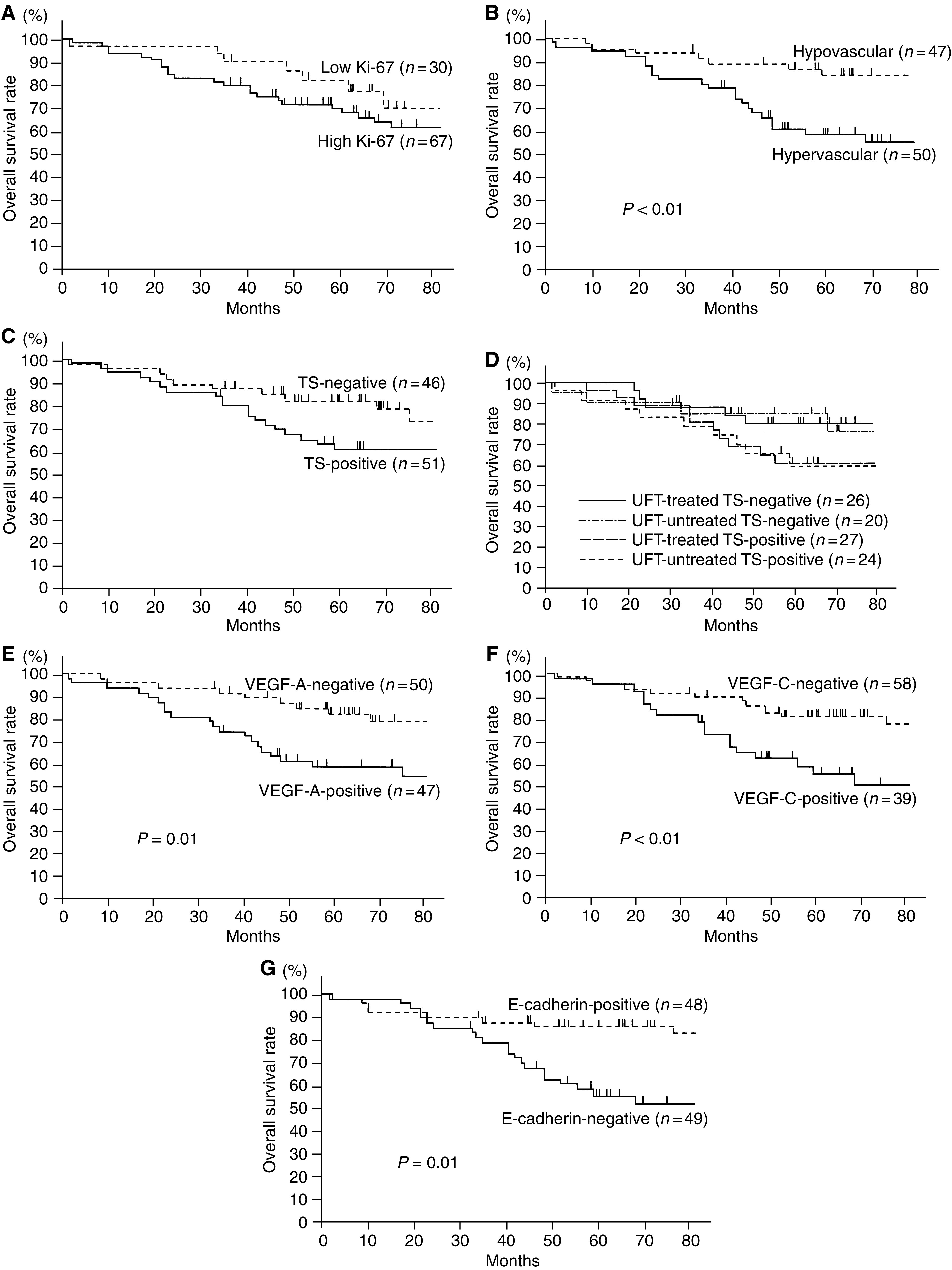
Overall survival of patients with stage I NSCLCs in relation to biological markers. (**A**) Ki-67 index, (**B**) tumour vascularity, (**C**) TS status, (**D**) TS status and UFT-based chemotherapy, (**E**) VEGF-A status, (**F**) VEGF-C status, and (**G**) E-cadherin status.

The 5-year survival rates of 97 patients with stage I NSCLCs in relation to biological markers are shown in Table 3. With respect to patients with stage I NSCLCs, there was no significant difference in the 5-year survival rates between patients with high Ki-67 tumours and those with low Ki-67 tumours (70.0 *vs* 82.2%, Figure 1A). In contrast, the 5-year survival rate of patients with hypervascular tumours was significantly lower than that of patients with hypovascular tumours (58.3 *vs* 83.5%, *P*<0.01, Figure 1B).

On the other hand, there was no significant difference in the 5-year survival rates between patients with TS-positive tumours and those with TS-negative tumours (60.7 *vs* 81.8%, Figure 1C). Regarding the TS status and UFT-based chemotherapy, there was no significant difference in the 5-year survival rates according to intratumoral TS status and UFT-based chemotherapy among stage I NSCLCs (Figure 1D). There was also no significant difference in the 5-year survival rates according to intratumoral TS status and UFT-based chemotherapy among stage I adenocarcinomas of the lung (72.2% in UFT-treated patients with TS-negative tumours, 77.2% in UFT-untreated patients with TS-negative tumours, 56.3% in UFT-treated patients with TS-positive tumours, and 66.7% in UFT-untreated patients with TS-positive tumours).

In contrast, the 5-year survival rate of patients with VEGF-A-positive tumours was significantly lower than that of patients with VEGF-A-negative tumours (58.6 *vs* 82.2%, *P*=0.01, Figure 1E). The 5-year survival rate of patients with VEGF-C-positive tumours was also significantly lower than that of patients with VEGF-C-negative tumours (54.9 *vs* 80.3%, *P*<0.01, Figure 1F). Furthermore, the 5-year survival rate of patients with E-cadherin-negative tumours was significantly lower than that of patients with E-cadherin-positive tumours (55.2 *vs* 85.3%, *P*=0.01, Figure 1G).

The multivariate analysis demonstrated that four variables, IMD (hazard ratio=3.34, *P*<0.01), VEGF-A status (hazard ratio=2.37, *P*=0.03), VEGF-C status (hazard ratio=2.10, *P*=0.03), and E-cadherin status (hazard ratio=2.30, *P*=0.03), were significant prognostic factors in patients with stage I NSCLCs (Table 4). The proportional hazards assumption was met adequately.

### Survival of patients with stage II–III NSCLCs in relation to biological markers

**Figure 2:**
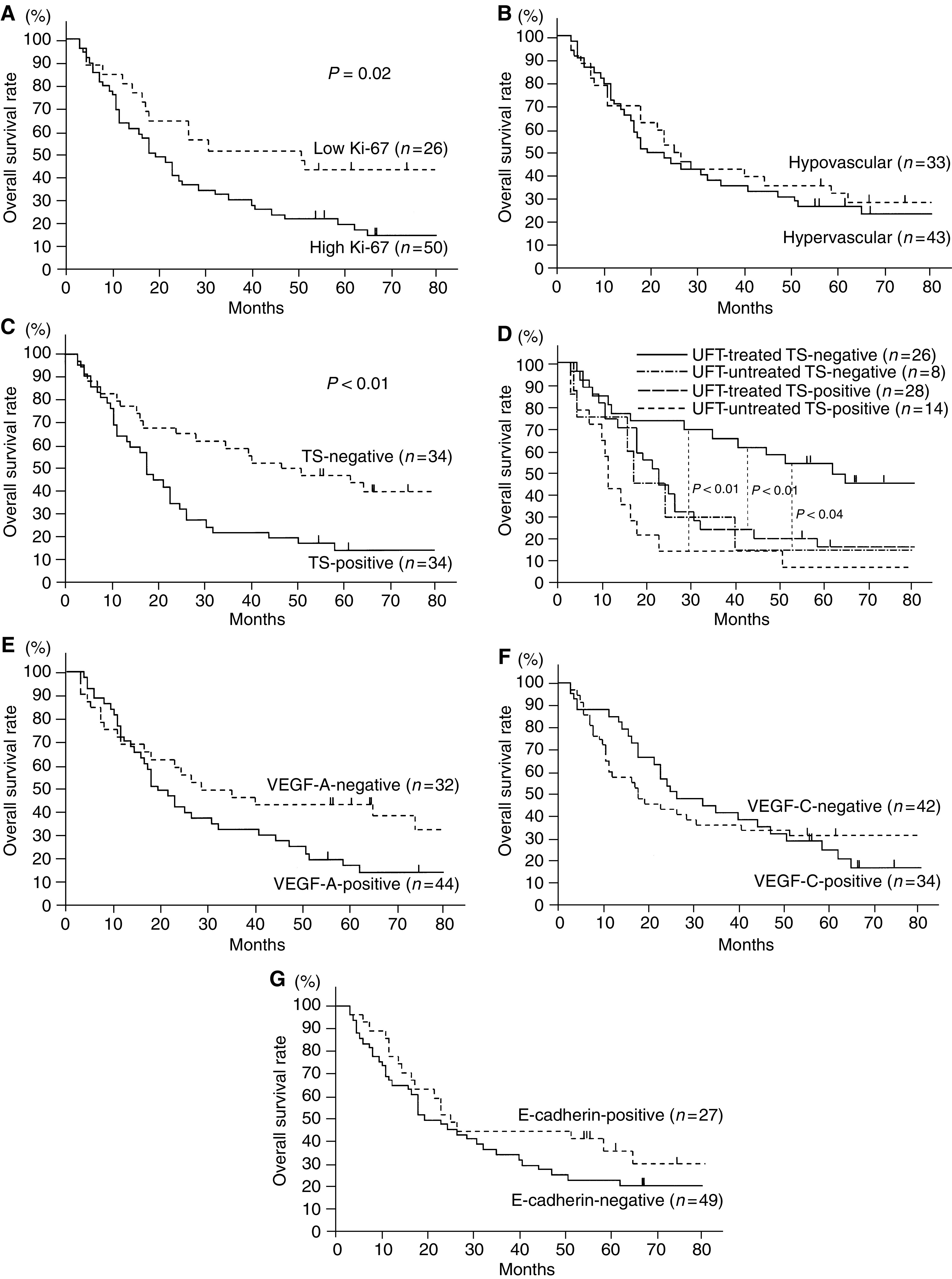
Overall survival of patients with stage II–III NSCLCs in relation to biological markers. (**A**) Ki-67 index, (**B**) tumour vascularity, (**C**) TS status, (**D**) TS status and UFT-based chemotherapy, (**E**) VEGF-A status, (**F**) VEGF-C status, and (**G**) E-cadherin status.

The 5-year survival rates of 76 patients with stage II–III NSCLCs in relation to biological markers are also shown in Table 3. With respect to patients with stage II–III (locally advanced) NSCLCs, the 5-year survival rate of patients with low Ki-67 tumours was significantly higher than that of patients with high Ki-67 tumours (43.2 *vs* 20.0%, *P*=0.02, Figure 2A). Especially, the 5-year survival rate of patients with low Ki-67 tumours was significantly higher than that of patients with high Ki-67 tumours among stage III (42.8 *vs* 16.7%, *P*=0.04). However, there was no difference in the 5-year survival rates between patients with hypovascular tumours and those with hypervascular tumours (31.1 *vs* 24.6%, Figure 2B).

Concerning TS status, the 5-year survival rate of patients with TS-negative tumours was significantly higher than that of patients with TS-positive tumours among stage II–III (45.8 *vs* 12.6%, *P*<0.01, Figure 2C). Especially, the 5-year survival rate of patients with TS-negative tumours was significantly higher than that of patients with TS-positive tumours among stage III (45.9 *vs* 8.7%, *P*<0.01). Regarding TS status and UFT-based chemotherapy, furthermore, the 5-year survival rates were 53.8% in UFT-treated patients with TS-negative tumours, 15.0% in UFT-untreated patients with TS-negative tumours, 15.2% in UFT-treated patients with TS-positive tumours, and 7.1% in UFT-untreated patients with TS-positive tumours (Figure 2D). In patients with stage II–III NSCLCs, the 5-year survival rate of UFT-treated patients with TS-negative tumours was significantly higher than those of any other patients (*P*=0.04, *P*<0.01, and *P*<0.01, respectively).

However, there was no difference in the survival of patients with stage II–III NSCLCs in relation to intratumoral expressions of VEGF-A, VEGF-C, or E-cadherin (Figure 2E–G).

The multivariate analysis also demonstrated that the Ki-67 proliferation index (hazard ratio=2.04, *P*=0.02) and TS status (hazard ratio=2.43, *P*<0.01) were significant prognostic factors in patients with stage II–III NSCLCs (Table 4). The proportional hazards assumption was met adequately.

## DISCUSSION

Recent studies on molecular biology in human cancers have revealed that many molecules affect various biological behaviours of malignant tumours. For example, the activation of oncogenes or the inactivation of tumour suppressor genes, such as K-ras mutation and p53 mutation, could initially cause malignant progression (Huang *et al*, 1998). In addition, reduced expressions of metastatic suppressor genes, such as E-cadherin (Sulzer *et al*, 1998; Liu *et al*, 2001), MRP-1/CD9, and KAI1/CD82 (Adachi *et al*, 1998), could induce tumour cells with high metastatic potential. In addition, tumour angiogenesis, affected by various angiogenetic factors such as the VEGF family (Dvorak *et al*, 1995), is associated with not only tumour growth but also tumour metastasis (Folkman, 1995).

The clinical use of these biological markers could be classified into categories as follows: detection of aggressive tumours with a poor prognosis among early stage tumours (Adachi *et al*, 1998; Liu *et al*, 2001), identification of less aggressive tumours with a relatively good prognosis among advanced stage tumours, and selection of effective adjuvant therapies including chemotherapy (made-to-order medicine) (Rosell *et al*, 2003; Vokes and Choy, 2003).

Many biological markers have been reported to be correlated with the survival of NSCLC patients. However, most biological markers associated with tumour angiogenesis and metastatic potential are correlated with the prognosis of early-stage NSCLCs (Adachi *et al*, 1998; Liu *et al*, 2001; Masuya *et al*, 2001). The present study also demonstrated that the survival of patients with stage I NSCLCs was significantly lower in patients with hypervascular tumours than in patients with hypovascular tumours. In addition, VEGF-C expression, another member of the VEGF family and reported to induce lymphangiogenesis (Skobe *et al*, 2001), was also associated with the prognosis of stage I patients, as reported previously (Kajita *et al*, 2001; Arinaga *et al*, 2003). Furthermore, previous clinical studies have shown that reduced expressions of several metastatic suppressor genes, including E-cadherin (Sulzer *et al*, 1998; Liu *et al*, 2001), MRP-1/CD9, and KAI1/CD82 (Adachi *et al*, 1998), are also associated with nodal metastases and a poor prognosis of patients with stage I NSCLCs. Although there were a few wide CIs for the hazard ratios in Table 4, these results might be partly because of factors like differing follow-up.

These results indicate that these biological markers associated with tumour angiogenesis or metastasis are useful for detecting aggressive tumours with high metastatic potential and a poor prognosis among early-stage tumours. In contrast, regarding the methods of surgical resection among stage I NSCLCs, there was no significant difference in the 5-year survival rates between patients treated with a lobectomy and patients treated with a segmentectomy or a wedge resection (75.3 *vs* 69.0%, *P*=0.18). Therefore, postoperative adjuvant chemotherapy might be necessary even in patients with stage I NSCLCs (Arriagada *et al*, 2004; Kato *et al*, 2004; Strauss *et al*, 2004; Winton *et al*, 2004), when tumours are hypervascular or have reduced expressions of metastatic suppressor genes.

On the other hand, these biological markers, including tumour angiogenesis, VEGF-A, VEGF-C, and E-cadherin expression, did not affect the prognosis of patients with stage II–III NSCLCs in the present study. Reductions of MRP-1/CD9 and KAI1/CD82 were also reported to not be correlated with the survival of locally advanced stage patients (Adachi *et al*, 1998). Thus, tumour angiogenesis and tumour metastatic suppressor genes are not clinical indicators for patients with locally advanced NSCLCs. These results urged us to perform the present study.

We evaluated the tumour proliferation rate using the Ki-67 proliferation index (Scagliotti *et al*, 1993). Ki-67 antibody recognises the nuclear antigen expressed during G1, S, G2, and M phases of the cell cycle and not during the resting (G0) phase. The present study has demonstrated that the Ki-67 proliferation index is one of the significant prognostic factors in patients with stage II–III NSCLCs. In contrast, the Ki-67 proliferation index was not associated with the prognosis of stage I patients.

Regarding TS, its expression is regulated by a polymorphic tandem repeat sequence in the 5′-terminal regulatory region of TS gene (Horie *et al*, 1995), and it is also regulated by several oncogenes and tumour suppressor genes, including E2F1, retinoblastoma, and p16/INK4 (DeGregori *et al*, 1995; Omura *et al*, 2000; Angus *et al*, 2002). Thymidylate synthase plays a central role in the biosynthesis of thymidylate, an essential precursor for DNA synthesis. Recent studies have revealed that TS exhibits oncogene-like activity. Its expression is associated with tumour cell proliferation (Navalgund *et al*, 1980), as demonstrated in the present study. Thymidylate synthase protein also downregulates p53 expression through TS protein–p53 mRNA interaction (Chu *et al*, 1999). In addition, TS can induce a transformed phenotype in mammalian cells (Rahman *et al*, 2004). The present study has demonstrated that the intratumoral TS expression is one of significant prognostic factors in patients with stage II–III NSCLCs.

Furthermore, the intratumoral TS activity has been reported to be related to 5-FU sensitivity (Beck *et al*, 1994). Several clinical studies have demonstrated that long-term oral UFT is effective postoperative adjuvant chemotherapy for NSCLC, especially in terms of patient quality of life (Wada *et al*, 1996; Kato *et al*, 2004). Many clinical studies on human cancers, including NSCLCs and gastrointestinal tumours, have shown that high TS expression is associated with 5-FU resistance and a poor outcome (Johnston *et al*, 1995; Yeh *et al*, 1998; Huang *et al*, 2000). In the present study, the 5-year survival rate of UFT-treated patients with TS-negative tumours was 53.8%, even in stage II–III, which was significantly higher than those of any other group (7.1–15.2%). A recent study has demonstrated that adjuvant chemotherapy with UFT improves the survival among patients with stage I adenocarcinoma of the lung (Kato *et al*, 2004). In the present study, however, there was no difference in the 5-year survival rates according to the intratumoral TS status and UFT-based chemotherapy among stage I NSCLCs or among stage I adenocarcinomas. These results might be partly due to the small number of patients studied.

These results suggest that the tumour proliferation rate and TS status are useful markers for identifying less aggressive tumours in locally advanced NSCLCs, and that TS expression is also a useful marker to evaluate responsiveness of UFT-based chemotherapy for these tumours. To our knowledge, there were few clinical reports to identify biological markers useful for the treatment of patients with locally advanced NSCLCs.

These findings indicate that evaluations of these markers, including the tumour proliferation index and target molecules for chemotherapy, would improve the clinical outcome of patients with locally advanced NSCLCs. Other than TS for UFT, epithelial growth factor receptor for Gefitinib (Mendelsohn and Baselga, 2003) and topoisomerase II for etoposide (Dingemans *et al*, 1999) might be useful target markers for chemotherapy in advanced NSCLC patients. In addition, such targeted therapies may lead to the development of made-to-order medicine, a new therapeutic strategy for cancer (Rosell *et al*, 2003; Vokes and Choy, 2003). Regarding target therapies, the present study was a retrospective clinical study with some limitations in its analysis. A further prospective randomised clinical study based on an evaluation of such biological markers, as the NCIC BR.19 study on adjuvant Gefitinib therapy (National Cancer Institute Central IRB protocol, 2002), should be performed.

## Figures and Tables

**Table 1:** Patient demographics

**Patient characteristics**	**Number**	**(%)**
Total number of patients	173	100
		
Age *(years)*		
Median	67	
Range	35–76	
		
*Gender*		
Male	116	67
Female	57	33
		
*Smoking habits*		
Nonsmoker	65	38
Smoker	108	62
		
*ECOG performance status*		
0	83	48
1	74	43
2	16	9
		
*Histology*		
Adenocarcinoma	101	58
Squamous cell carcinoma	58	34
Large-cell carcinoma	14	8
		
*Pathological stage*		
I	97	56
II	17	10
III	59	34
		
*Method of surgical resection*		
Pneumonectomy	16	9
Lobectomy	133	77
Segmentectomy	3	2
Wedge resection	21	12
		
*Chemotherapy*		
MVP	76	44
Neoadjuvant therapy	49	28
Postoperative adjuvant therapy	27	16
UFT	107	62
		
Radiotherapy	18	10

ECOG=Eastern Cooperative Oncology Group; MVP=mitomycin/vinblastin/cisplatin; UFT=a combination of tegafur and uracil.

**Table 2:** Distribution of biological markers in 173 NSCLC patients according to clinicopathological characteristics

		**High Ki-67**		**Hypervascular**		**TS-positive**		**VEGF-A-positive**		**VEGF-C-positive**		**E-cadherin-negative**	
**Characteristics**	***n***	**(%)**	***P*-value**	**(%)**	***P*-value**	**(%)**	***P*-value**	**(%)**	***P*-value**	**(%)**	***P*-value**	**(%)**	***P*-value**
*Smoking*													
Nonsmoker	65	50.8	<0.01	61.5	0.15	53.8	0.98	49.2	0.49	38.5	0.44	55.4	0.79
Smoker	108	77.8		49.1		53.7		54.6		44.4		57.4	
													
*Tumour status*													
T1, T2	125	68.8	0.59	52.0	0.45	50.4	0.15	51.2	0.55	41.6	0.79	53.6	0.19
T3, T4	48	64.6		58.3		62.5		56.3		43.8		64.6	
													
*Nodal status*													
N0	120	65.0	0.26	53.3	0.86	51.7	0.40	47.5	0.04	42.5	0.90	50.8	0.02
N1, N2	53	73.6		54.7		58.5		64.2		41.5		69.8	
													
*Pathological status*													
Stage I	97	69.1	0.39	51.5	0.54	52.6	0.39	48.5	0.21	40.2	0.78	50.5	0.09
Stage II	17	52.9		47.1		41.2		29.4		41.2		76.5	
Stage III	59	69.5		59.3		59.3		66.1		45.8		61.0	
													
*Histology*													
Adenocarcinoma	101	56.4	<0.01	65.3	<0.01	43.6	<0.01	54.5	0.70	45.5	0.22	52.5	0.06
Squamous cell carcinoma	58	87.9		32.8		72.4		51.7		41.4		56.9	
Large-cell carcinoma	14	64.3		57.1		50.0		42.9		21.4		85.7	
*Neoadjuvant MVP chemotherapy*													
Without neoadjuvant MVP	124	70.9	0.19	52.4	0.69	53.2	0.95	52.4	0.93	43.5	0.68	54.0	0.35
With neoadjuvant MVP	49	59.2		57.1		55.1		53.1		38.8		63.3	
													
Total number of patients	173	67.6		53.8		53.8		52.6		42.2		56.6	

NSCLC=non-small-cell lung cancer; TS=thymidylate synthase; VEGF=vascular endothelial growth factor; MVP=mitomycin/vinblastin/cisplatin.

**Table 3:** The 5-year survival rate of 173 NSCLC patients in relation to biological markers and pathological stage

	**Stage I**		**Stage II**		**Stage III**		**Stage II–III**		**Total**	
**Characteristics**	**(%)**	***P*-value**	**(%)**	***P*-value**	**(%)**	***P*-value**	**(%)**	***P*-value**	**(%)**	***P*-value**
*Ki-67 index*										
Low	82.2	0.57	43.8	0.45	42.8	0.04	43.2	0.02	67.0	0.01
High	70.0		33.3		16.7		20.0		44.1	
										
*IMD*										
Hypovascular	83.5	<0.01	50.8	0.30	25.7	0.60	31.1	0.82	62.3	0.02
Hypervascular	58.3		25.0		23.6		24.6		43.1	
										
*TS status*										
Negative	81.8	0.26	45.0	0.23	45.9	<0.01	45.8	<0.01	66.3	<0.01
Positive	60.7		28.6		8.7		12.6		39.2	
										
*VEGF-A status*										
Negative	82.2	0.01	55.6	0.20	35.0	0.75	42.6	0.37	66.5	<0.01
Positive	58.6		0.0		19.3		16.9		38.7	
										
*VEGF-C status*										
Negative	80.3	<0.01	40.0	0.91	27.5	0.75	30.3	0.88	59.2	0.03
Positive	54.9		34.3		21.6		24.3		40.6	
										
*E-cadherin status*										
Positive	85.3	0.01	50.0	0.93	32.6	0.15	35.6	0.28	67.5	<0.01
Negative	55.2		33.8		18.6		22.6		38.9	

NSCLC=non-small-cell lung cancer; IMD=intratumoral microvessel density; TS=thymidylate synthase; VEGF=vascular endothelial growth factor; MVP=mitomycin/vinblastin/cisplatin; E-cadherin=epithelial cadherin.

**Table 4:** Multivariate regression analyses in predicting survival of NSCLC patients

	**Stage I**			**Stage II, Stage III**		
**Biological markers**	**Hazard ratio**	**95% CI**	***P*-value**	**Hazard ratio**	**95% CI**	***P*-value**
Ki-67 index	1.75	0.73–4.21	0.20	2.04	1.09–3.80	0.02
IMD	3.34	1.30–7.86	<0.01	1.38	0.75–2.54	0.28
TS status	1.31	0.57–2.98	0.51	2.43	1.34–4.42	<0.01
VEGF-A status	2.37	1.07–5.24	0.03	1.27	0.69–2.35	0.43
VEGF-C status	2.10	1.01–4.37	0.03	0.94	0.54–1.64	0.83
E-cadherin status	2.30	1.03–5.16	0.03	1.50	0.85–2.63	0.15

NSCLC=non-small-cell lung cancer; CI=confidence interval; IMD=intratumoral microvessel density; TS=thymidylate synthase; VEGF=vascular endothelial growth factor; MVP=mitomycin/vinblastin/cisplatin; E-cadherin=epithelial cadherin.
